# The *ToxAvapA* Toxin-Antitoxin Locus Contributes to the Survival of Nontypeable *Haemophilus influenzae* during Infection

**DOI:** 10.1371/journal.pone.0091523

**Published:** 2014-03-12

**Authors:** Dabin Ren, Alexis A. Kordis, Daniel E. Sonenshine, Dayle A. Daines

**Affiliations:** 1 Department of Research, Rochester General Hospital Research Institute, Rochester, New York, United States of America; 2 Department of Biological Sciences, Old Dominion University, Norfolk, Virginia, United States of America; Indian Institute of Science, India

## Abstract

Nontypeable *Haemophilus influenzae* (NTHi) is an opportunistic pathogen that is a common cause of acute and recurrent mucosal infections. One uncharacterized NTHi toxin-antitoxin (TA) module, NTHI1912-1913, is a ***h***ost ***i***nhibition of ***g***rowth (*higBA*) homologue. We hypothesized that this locus, which we designated *toxAvapA*, contributed to NTHi survival during infection. We deleted *toxAvapA* and determined that growth of the mutant in defined media was not different from the parent strain. We tested the mutant for persistence during long-term *in vitro* co-culture with primary human respiratory tissues, which revealed that the Δ*toxAvapA* mutant was attenuated for survival. We then performed challenge studies using the chinchilla model of otitis media and determined that mutant survival was also reduced *in vivo*. Following purification, the toxin exhibited ribonuclease activity on RNA *in vitro*, while the antitoxin did not. A microarray comparison of the transcriptome revealed that the tryptophan biosynthetic regulon was significantly repressed in the mutant compared to the parent strain. HPLC studies of conditioned medium confirmed that there was no significant difference in the concentration of tryptophan remaining in the supernatant, indicating that the uptake of tryptophan by the mutant was not affected. We conclude that the role of the NTHi *toxAvapA* TA module in persistence following stress is multifactorial and includes effects on essential metabolic pathways.

## Introduction

Nontypeable *Haemophilus influenzae* (NTHi) are pleomorphic Gram-negative bacteria that reside in the human upper respiratory tract as commensals. However, these organisms are also responsible for a number of mucosal diseases, including chronic bronchitis, exacerbations of chronic obstructive pulmonary disease, and acute and chronic otitis media (OM). Recurrent OM caused by NTHi is common, and those infections that recur less than two weeks after the completion of antimicrobial therapy have been shown to largely be due to the same strain of bacteria, suggesting a subpopulation of persister cells [1], [2]. Toxin-antitoxin (TA) gene pairs have been found in nearly all bacterial genomes sequenced to date, and type II loci encode a protein toxin and antitoxin that form a nontoxic complex upon translation which auto-represses the cognate promoter [3]. Under stressful environmental conditions such as nutrient limitation, antibiotic therapy, or oxidative stress, the labile antitoxin is degraded by intracellular proteases and the more stable toxin is freed to facilitate growth arrest, often via mRNA degradation [4], [5]. The ability to induce a state of dormancy increases microbial fitness by decreasing nutrient requirements and the metabolic burden. As well, this persister state facilitates nonspecific antibiotic tolerance in the microorganism, as most antimicrobials target essential cellular functions necessary for bacterial growth and replication [6].

TA modules have been divided into several families, one of which is the ***h***ost ***i***nhibition of ***g***rowth (*higBA*) gene pair [7]. Most TA operons are organized such that the antitoxin is transcribed first, followed by the toxin, and the *higBA* locus is unusual in that the toxin gene precedes the antitoxin. Originally identified on the *Proteus vulgaris* Rts1 plasmid [7], the *higBA* module has been found in the chromosomes of a number of pathogens, including *Vibrio cholerae*, *Mycobacterium tuberculosis*, *Pseudomonas aeruginosa*, *Yersinia pestis*, and *Acinetobacter baumannii* [8]–[12]. NTHi strains contain three TA gene pair families, and one of these is a *higBA* homologue. In previous work, we have shown that two of the TA loci in NTHi exert significant effects on the organism's ability to sustain an infection, both *in vitro* and *in vivo* [13]. In this study, we investigated the hypothesis that the *higBA* homologue in the NTHi strain 86-028NP (NTHI1912-1913, designated *toxAvapA*), also played a role in the survival of this human-adapted pathogen. We found that the *toxAvapA* locus enhanced the ability of NTHi to persist during *in vitro* and *in vivo* infections.

## Materials and Methods

### Bacterial Strains and Plasmids

The bacterial strains and plasmids used in these studies are listed in Table 1. *E. coli* strains were grown in LB broth or agar with or without 30 μg/ml kanamycin or 100 μg/ml ampicillin, as required. NTHi strains were grown in brain heart infusion supplemented with 10 μg/ml heme-histidine and 10 μg/ml β-NAD (sBHI) broth or agar. *E. coli* strain BL21(DE3) was used to overexpress *toxAvapA* for protein purification prior to use in ribonuclease activity assays. NTHi transformants were selected on chocolate agar plates with 25 μg/ml spectinomycin, and were routinely cultured at 37°C with 5% CO_2_.

**Table 1:** Bacterial strains and plasmids used in this study.

Strain	Description	Source
DH5α	F^−^ Φ80*lacZ*ΔM15 Δ (*lacZYA-argF*) U169 *recA1 endA1 hsdR17* (rK^−^, mK^+^) *phoA supE44* λ– *thi-1 gyrA96 relA1*	Lab collection
BL21(DE3)	F^−^ *ompT hsdSB*(rB^−^, mB^−^) *gal dcm* (DE3)	NEB
86-028NP	Nontypeable *Haemophilus influenzae* strain isolated from the nasopharynx of a child being treated for chronic otitis media	[14]
Δ*toxAvapA*	Strain 86-028NP with the *toxAvapA* locus deleted.	This work
**Plasmid**	**Description**	**Source**
pET24b	Bacterial vector for expressing polyhistidine-tagged proteins	Novagen
pBluescript SK^+^	Bacterial cloning vector	Lab collection
pDD849	pBluescript SK^+^ with the 5′ arm of the *toxAvapA* plasmid.	This work
pDD851	pDD849 with the 3′ arm of the *toxAvapA* plasmid.	This work
pDD857	pDD851 with the spectinomycin resistance cassette.	This work
pDD912	pET24b with the *toxAvapA* locus for protein expression.	This work

### Construction of a Δ*toxAvapA* Mutant

The Δ*toxAvapA* mutation construct was assembled by amplifying genomic DNA (gDNA) from NTHi strain 86-028NP [14] using Phusion FLASH high fidelity polymerase (Thermo Fisher Scientific, Waltham, MA USA), which results in blunt-ended amplicons, and primers with engineered restriction sites (underlined) KpnToxFor (5′-AAAAGGTACCGGCGAGTGCAATCAC-3′) and ClaToxRev (5′-GATAATCGATTCTAAAATGCTCACG-3′). The 1000 bp PCR product was cut with *KpnI* and *ClaI* and fused with *KpnI*/*ClaI-*digested pBluescript SK^+^, and designated as pDD849. The second arm of the construct was made by amplifying 86-028NP gDNA using the primers 86APstFor (5′-TCTACTGCAGCAATGTAATTTGAGC-3′) and 86XbaVapARev (5′-GTAATCTAGAGAAGATCCAACCAGC-3′). The *XbaI*-digested 883 bp amplicon was ligated to pDD849 digested with *Smal* and *XbaI*, resulting in pDD851. A 1200 bp product which contained a spectinomycin resistance cassette was PCR-amplified from pDD872 using the primer set pUC4 For (5′-TTCGCTATTACGCCAGCTGG-3′) and pUC4 Rev (5′-GCCGATTCATTAATGCAGCTG-3′), cut with *EcoRI* and ligated to pDD851 digested with *EcoRI*, generating pDD857. A 3083 bp fragment from pDD857 was amplified with KpnToxFor and 86XbaVapARev primers and used to transform NTHi strain 86-028NP using the M-IV method as previously described by Herriott *et al*. [15]. Colonies resistant to 25 μg/ml spectinomycin were selected and the deletion of *toxAvapA* was identified by PCR and verified by DNA sequencing of both strands.

### Growth Curves of the Δ*toxAvapA* Mutant and the Parent Strain

The 86-028NP parent strain or the Δ*toxAvapA* mutant were re-suspended from sBHI agar plates grown for 18 hours at 37°C in 5% CO_2_ into fresh defined media [16] at an OD_600_ of ∼0.1, then 200 microliters of each re-suspension was placed in duodecuplicate into a sterile non-treated flat-bottomed 96 well plate (#351172, BD Biosciences, Bedford, MA, USA). Empty wells were filled with 200 microliters of sterile water to decrease evaporation, and the plate was covered with sterile gas permeable sealing film (#9123-6100, USA Scientific, Ocala, FL, USA). The plate was incubated for 15 hours with shaking at 35°C in a Multiskan FC spectrophotometer (ThermoFisher Scientific, Waltham, MA, USA), and the OD_595_ was read hourly. Three biological replicates were performed and analyzed by the repeated measures analysis of variance (RMANOVA).

### NTHi Invasion Assays in the EpiAirway Tissue Model

EpiAirway tissues (MatTek, Ashland, MA USA) were inoculated with either 10^6^ NTHi strain 86-028NP (n = 6) or Δ*toxAvapA* mutant (n = 6) suspended in pre-warmed Dulbecco's phosphate-buffered saline with calcium and magnesium (DPBS^+^) in a total volume of ≤25 microliters onto the apical surface. Tissues were incubated at 37°C in 5% CO_2_ until harvest at day 2, 4, or 8 after infection. Tissues were maintained by washing the apical surface with 200 μl of DPBS^+^ and the basal media was changed using 1 ml of MatTek airway serum-free medium on a daily basis. On the day of harvest, inserts were washed with DPBS^+^ three times, and then 250 μl of RPMI 1640 supplemented with 100 μg/ml gentamicin (MP Biomedical, Solon, OH USA) was added to the apical side, with 1 ml added to the basal side to kill any external cell-associated bacteria. After incubation at 37°C in 5% CO_2_ for one hour, each insert was washed three times with DPBS without calcium and magnesium (DPBS^−^) and 250 μl of sterile 1% saponin (Sigma-Aldrich, St. Louis, MO USA) in DPBS^−^ was added to the apical surface. The inserts incubated at 37°C with 5% CO_2_ for 10 minutes. The tissue was then physically disrupted from the membrane and collected followed by the addition of 250 μl DPBS^−^ to the apical surface and the remaining tissue was collected. Total tissue collection was confirmed by microscopy of the insert. The volume of the collected tissue suspension was increased to 1 ml with DPBS^−^. Each sample was then vortexed vigorously to de-aggregate the cells and serially diluted using sterile PBS. Aliquots were drop-plated onto chocolate agar plates to enumerate viable internalized bacteria.

### Survival of NTHi in the Chinchilla Otitis Media Model

#### Ethics Statement:

Guidelines published in the Guide for the Care and Use of Laboratory Animals of the National Institutes of Health were followed for all animal handling and husbandry. The protocol #A1110011 was approved by the Mercer University Institutional Animal Care and Use Committee (Animal Welfare Assurance Number: A3725-01). All surgery was performed under isoflurane anesthesia, and all efforts were made to minimize suffering.

Adult female chinchillas (400–600 g) were purchased from a commercial supplier and allowed to acclimate in the vivarium for one week prior to bacterial challenge. On the day of challenge, each animal was examined by otoscopy for signs of middle ear infection. Both the wild-type and the Δ*toxAvapA* mutant strain from frozen stocks were plated on chocolate agar and incubated at 37°C with 5% CO_2_ for 18 h. Sterile DPBS with 0.1% gelatin (Sigma-Aldrich, St. Louis, MO USA) (DPBS-G) was inoculated with the bacteria to a concentration of 1.0×10^4^ CFU/ml and 100 μl of this suspension was loaded into 1 cc syringes. After chinchillas were anesthetized by isoflurane inhalation, 100 μl (∼10^3^ CFU) of bacteria was injected into the superior bullae of each animal (n = 4 animals with 8 middle ears per challenge strain). On day 4 post-challenge, chinchillas were humanely euthanized by cardiac exsanguination and middle ear fluid was collected. Each middle ear was then washed with 1 ml DPBS-G. The wash fluid was collected, serially diluted and drop-plated on chocolate agar for viable bacterial counts after 18 h incubation at 37°C with 5% CO_2_.

### Histology of the Chinchilla Middle Ear

Chinchilla bullae were dissected and fixed with 10% neutral buffered formalin for 24 hours at room temperature. The bullae were then decalcified with 5% formic acid for 48 hours and cut at the midline in the sagittal plane. Each half was embedded in paraffin and step sections of the distal portion were mounted onto slides, stained with hematoxylin-eosin (H&E) and examined to determine successful establishment of infection.

### Total RNA Isolation

Cultures of the wild-type and Δ*toxAvapA* mutant were grown in 35 ml of defined media [16] in baffled flasks at 35°C with shaking. Once the OD_600_ reached ∼0.4, five ml of culture was added to 5 ml of RNAlater Solution (Ambion, Life Technologies, Grand Island, NY USA) and stored at 4°C until processed. The experiment was repeated in triplicate and all repetitions were processed within 48 hours of collection. Total RNA was isolated using the ChargeSwitch Total RNA Cell Kit (Invitrogen, Life Technologies, Grand Island, NY USA) according to the manufacturer's directions, with the modification of a vial change and an extended DNAse incubation step (30 min at 37°C). Following purification, the concentration of the total RNA was determined by a Nanodrop 2000c spectrophotometer (Thermo Scientific Inc., Pittsburgh, PA USA) and aliquots were prepared and stored at −80°C. Prior to cDNA synthesis, PCR using an aliquot of total RNA as the template was performed to confirm that each preparation was free of contaminating genomic DNA.

### Transcriptome Study

The High Capacity cDNA Reverse Transcriptase kit (Applied Biosystems, Life Technologies, Grand Island, NY USA) was used to synthesize cDNA from a pool of total RNA that represented equal concentrations of three independent RNA isolations per strain. The cDNA reactions were performed in 100 μl aliquots according to the manufacturer's directions, with the modification that the cDNA was treated with RNase A for 30 min at 35°C following completion of the protocol. The cDNA was then column-purified using the MinElute kit (Qiagen, Valencia, CA USA) and the concentration of the cDNA was determined by a Nanodrop 2000c spectrophotometer. Approximately 2 μg of cDNA from either the wild-type or the Δ*toxAvapA* mutant was labeled and hybridized to a custom 4×72 K Nimblegen microarray by the Florida State University-Nimblegen Microarray Facility (Tallahassee, FL USA). Data was analyzed using the ArrayStar Version 5.1.0 build 114 software (DNASTAR Inc., Madison, WI USA). Validation of microarray results was done by qPCR on selected genes using a MiniOpticon real-time thermocycler (Bio-Rad, Hercules, CA USA). Briefly, 25 μl reactions were prepared with 12.5 μl of 2X Power SYBR Green Master Mix (Life Technologies, Grand Island, NY USA), 400 nM of each gene-specific primer set, and 5 μl of a 1∶10 dilution of the cDNA used in the microarray study, as well as an independent replicate performed as an additional control. Relative expression was calculated by the comparative C_t_ method (2^−ΔΔCt^) using wild-type cDNA as the calibrator and the expression of a DNA gyrase subunit gene, *gyrA*, as the endogenous control.

### High Performance Liquid Chromatography (HPLC) of Conditioned Medium

HPLC was used to determine the tryptophan concentrations in conditioned medium from the wild-type and the Δ*toxAvapA* mutant. Briefly, each strain was inoculated in defined medium at an OD_600_ of ∼0.1 and grown with shaking at 35°C to an OD_600_ of 0.4. Five milliliters of each culture was pelleted and the supernatants were passed through 3 kDa MWCO filters (Vivaspin 500, GE Healthcare, Pittsburgh, PA USA). HPLC was done on pooled filtrates representing three replicates of each strain using a Waters model 680 automated gradient controller, two model 510 pumps, a Rheodyne model 7725i manual injector (Waters, Milford, MA USA) and a Shimadzu model SPD-M10A photodiode array detector (Shimadzu Instrument Co., Columbia, MD USA). The column was a Sorbax Eclipse AAA column, 150 mm×4.6 mm i.d. reversed phase C-18 stainless steel column containing 3.5 μm size silica particles (Agilent Technologies, Santa Clara, CA USA). The solvents used were (A) 40 mM NH_4_H_2_PO_4_ solution (10.4 pH) and (B) acetonitrile: methanol, 60∶40. All solvents were HPLC grade. Run conditions were: 95% A:5% B for 8 min; then 80% A: 20% B to 20 min; 75% A: 25% B at 30 min; 95% A: 5% B at 40 min; 100% A at 42 min; and, finally, return start conditions after 45 min [17]. Flow rates were 0.75 mls/min. Authentic tryptophan (Sigma Aldrich, St. Louis, MO USA) was suspended in sterile distilled water at 5 mg/ml. Pre-column derivatization was done with *o*-phthaldialdehyde/mercapto-ethanol (OPA) (Sigma Aldrich, St. Louis, MO USA). Samples ranging from 5, 10, 25 or 50 μg of authentic tryptophan were injected into the HPLC system to determine elution times and spectral characteristics for this amino acid standard (and distinct from background peaks due to OPA alone, which were excluded). Subsequently, samples of conditioned media from NTHi wild-type and mutant were derivatized, injected into the HPLC and the co-elution times, spectral characteristics and differences in the amounts (milli-absorbance units) of the putative sample tryptophan peak were determined. To confirm the identity of the elution times, several samples of the conditioned media also were spiked with authentic tryptophan.

Tryptophan-specific peaks were collected using a Pharmacia Frac-100 fraction collector (Amersham Biosciences, Piscataway, NJ USA), lyophilized in a FreeZone Plus 2.5 L lyophilizer (Labconco, Kansas City, MO USA) and resuspended in sterile distilled water. The lyophilized fractions were then assayed by thin layer chromatography (TLC) using 10×20 cm silica gel/TLC cards (Sigma Aldrich, St. Louis, MO USA) and eluted for 2 h with n-butanol:acetic acid:water 3∶1∶1 (by volume). The dried card was then examined by UV fluorescence at 254 nm and stained with ninhydrin (0.1 g ninhydrin, 100 ml n-butanol, 0.5 ml acetic acid) followed by gentle heating. Tryptophan in the fractions was confirmed by co-elution with the authentic standard.

### ToxA and VapA Protein Expression and Purification

The *toxAvapA* operon from NTHi strain 86-028NP was cloned into pET24b by amplifying gDNA with the following primer pair, 86ToxBamFor (5′-GAGAGGATCCGATGTTTAATTTAAAGCG-3′) and ASacRev (5′-GGCTGAGCTCACATTGCAAATGTAG-3′). The PCR product was cut with *BamHI* and *SacI* and fused with *SacI* and *BamHI*-digested pET24b, generating pDD912. Following DNA sequencing, the plasmid pDD912 was transformed into *E. coli* BL21(DE3) strain and grown in 30 ml of LB broth supplemented with 30 μg/ml kanamycin with shaking in baffled flasks at 35°C. When the OD_600_ reached ∼0.4, 1 mM IPTG was added and the culture was incubated for an additional 3 hours. Ten ml cell pellets were isolated, subjected to 3 freeze/thaw cycles, resuspended in 5 ml of BugBuster solution (1× BugBuster, 100 mM HEPES, 1× Halt EDTA-free protease inhibitor, 10 μl DNAse) and rotated at room temperature for 30 minutes. The solution was then sonicated with 1.5 second pulses at 10% power for 12 cycles using a Branson Sonifier (Branson UltraSonics Co. Ltd, Shanghai, P.R. China) equipped with a microtip. The resulting lysate contained the ToxAVapA protein complex. The MagneHis™ protein purification system (Promega Corp., Madison, WI USA) was used to bind polyhistidine-tagged VapA with the following modifications. The lysate with the ToxAVapA complex bound to Ni^+^ magnetic beads was washed twice with wash buffer (100 mM HEPES (pH 7.5), 150 mM NaCl, 10 mM imidazole). The bead pellet was then resuspended in 200 μl of denaturing buffer (100 mM HEPES (pH 7.5) and 8 M urea) and incubated at room temperature for 15 min on a rotator. The supernatant was collected and removed, representing purified denatured ToxA protein. The polyhistidine-tagged VapA protein was then recovered from the Ni^+^ beads with denaturing elution buffer (100 mM HEPES, 8 M urea and 500 mM imidazole) according to the manufacturer's protocol. Protein concentrations of both ToxA and VapA were determined by Bradford assay and protein isolation was confirmed by SDS-PAGE followed by staining with GelCode Blue (Thermo Scientific Inc., Pittsburgh, PA USA).

To re-nature the purified proteins, two hundred microliter aliquots of denatured ToxA or VapA were placed into individual Slide-A-Lyzer® Mini Dialysis 3,500 Da MWCO tubes (Thermo Scientific Inc., Pittsburgh, PA USA) and dialyzed for 18 hours at 4°C against 25 ml of either acidic refolding buffer (50 mM Na acetate (pH 4.0), 100 mM KCl, 200 mM arginine, 50 mM NDSB-201 (3-(1-Pyridino)-1-propane sulfonate) and 50 mM NDSB-256 (Dimethylbenzylammonium propane sulfonate) for ToxA (pI 9.1), or alkaline refolding buffer (50 mM Tris (pH 8.0), 100 mM KCl, 200 mM arginine, 50 mM NDSB-201 (3-(1-Pyridino)-1-propane sulfonate) and 50 mM NDSB-256 (Dimethylbenzylammonium propane sulfonate) for VapA (pI 6.7) [18]. These buffers were then replaced with fresh dialysis buffer containing 10% glycerol, and dialyzed at 4°C for an additional 4 h. The re-natured proteins were concentrated using 3.0 kDa MWCO centrifugal filters (Vivaspin 500, GE Healthcare, Pittsburgh, PA USA) and assayed via the Bradford method to determine total protein concentration.

### RNase Activity Assays

The RNaseAlert® substrate (IDT, Coralville, Iowa USA) was used to determine re-natured ToxA and VapA RNase activity. ToxA and VapA were diluted to 0.05 mg/ml in 1× RNaseAlert proprietary buffer and acclimated to room temperature for 30 min. Eighteen pmol of each protein dilution was added to 25 μl RNaseAlert substrate in triplicate wells of a black clear-bottomed 96-well plate (Corning #3340, Tewksbury, MA USA) and incubated at 37°C for 1 h. The negative control was an identical volume of protein renaturation buffer, and the positive control was 18 pmol bovine pancreatic RNase A (Thermo Scientific Fermentas, Pittsburgh, PA USA). Fluorescence (excitation 485 nm, emission 520 nm) was measured using a FLUOstar OPTIMA plate reader (BMG Labtech, Ortenberg, Germany).

### ToxA Kinetic Studies

To determine the initial reaction progress, increasing amounts of the RNaseAlert substrate (5, 10, and 12.5 pmol) were incubated with 18 pmol of renatured ToxA in a final volume of 30 microliters. The reactions were incubated at 37°C in a MiniOpticon real-time thermocycler (Bio-Rad, Hercules, CA USA) and fluorescence was measured every 30 seconds on the fluorescein (FAM) channel for the first 6 minutes. The time zero measurement was subtracted from each well. A separate set of reactions using 5 pmol RNaseAlert substrate and 18 pmol renatured ToxA were incubated at 37°C in a MiniOpticon and fluorescence was measured every 30 seconds for 30.5 minutes to allow the reaction to approach completion.

### Data Analysis

All data are presented as the mean ± standard deviation. The significance of any differences between means was determined using Student's *t* test, analysis of variance (ANOVA) followed by the Tukey-Kramer test, or repeated measures ANOVA (RMANOVA). A *p* value of ≤0.05 was considered statistically significant.

## Results and Discussion

### Growth Dynamics in Defined Media

**Figure 1:**
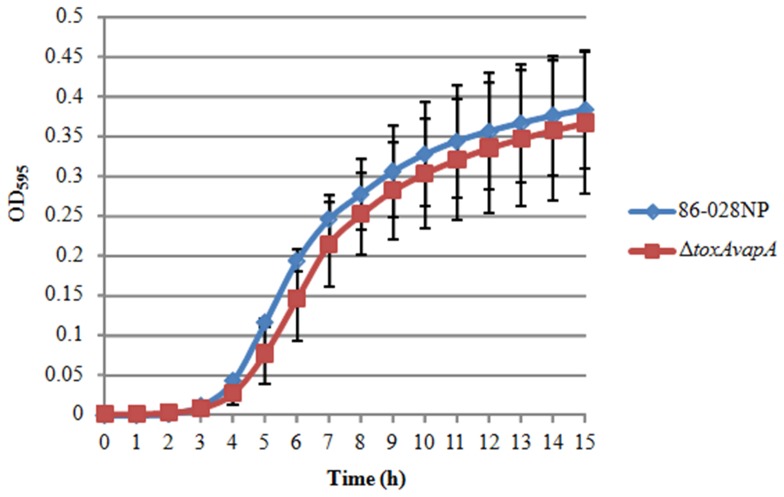
Growth curve of the Δ*toxAvapA* mutant and parent strain in defined media. No significant difference was found between the growth of the parent and mutant strains (n = 3 in duodecuplicate).

We deleted the entire *toxAvapA* operon in NTHi strain 86-028NP, and following confirmation of the deletion, we performed growth curves in defined media to determine whether there were any growth defects in the Δ*toxAvapA* mutant as compared to the parent strain. We found that there were no significant differences by repeated measures ANOVA between the growth of the wild-type versus the mutant over a 15 hour time period in defined media (Figure 1). These data suggest that any observed differences in survival of the mutant *in vitro* or *in vivo* would not be attributable to a fundamental reduction in the organism's ability to replicate over the growth cycle.

### Co-culture in Primary Human Tissues

NTHi normally reside in the human upper respiratory tract in close association with the respiratory epithelium. To determine any effects of the *toxAvapA* deletion on the ability of NTHi to survive during long-term co-culture with primary human respiratory epithelial tissues, we used the EpiAirway™ model (MatTek, Ashland, MA USA). These 3-dimensional, highly differentiated tissues from normal human donors are metabolically and mitotically active and have the capacity to survive at the air-liquid interface for weeks. Further, the proprietary media supplied with the tissues is serum-free. This allowed us to perform long-term co-culture of these tissues with both the wild-type and the Δ*toxAvapA* mutant. We inoculated tissues with ∼1.0×10^6^ CFU of each strain, and at days 2, 4, and 8 post-infection, we recovered the viable gentamicin-resistant (internalized) bacteria that persisted within the tissues over time (Table 2).

**Table 2:** Number of viable gentamicin-resistant internalized bacteria that survived over time in the EpiAirway human tissue model (n = 6).

Day	CFU/ml WT (±SD)	CFU/ml Δ*toxAvapA* (±SD)	*p* value	% WT
2	8.9E+05 (1.6E+06)	6.2E+04 (5.9E+04)	0.047	7.0
4	7.3E+05 (4.2E+05)	4.3E+05 (1.3E+05)	0.038	58.9
8	2.5E+06 (1.4E+06)	9.5E+05 (6.8E+05)	0.014	38.2

In all cases, the Δ*toxAvapA* mutant was attenuated for survival compared to the parent strain. This trend was significant at all time points, but the observed effect of the *toxAvapA* deletion was most substantial at the first harvest (2 days post-inoculation), at which only 7.0% of the wild-type numbers were recovered. This percentage increased at day 4 to 58.9% but declined again by day 8 to 38.2%, indicating that the mutant was unable to survive at the levels of the wild-type strain over the entire experimental period. This is the first report, to our knowledge, of the contribution of *toxAvapA* to the survival of NTHi in primary human tissues.

### Chinchilla Model of Otitis Media

**Figure 2:**
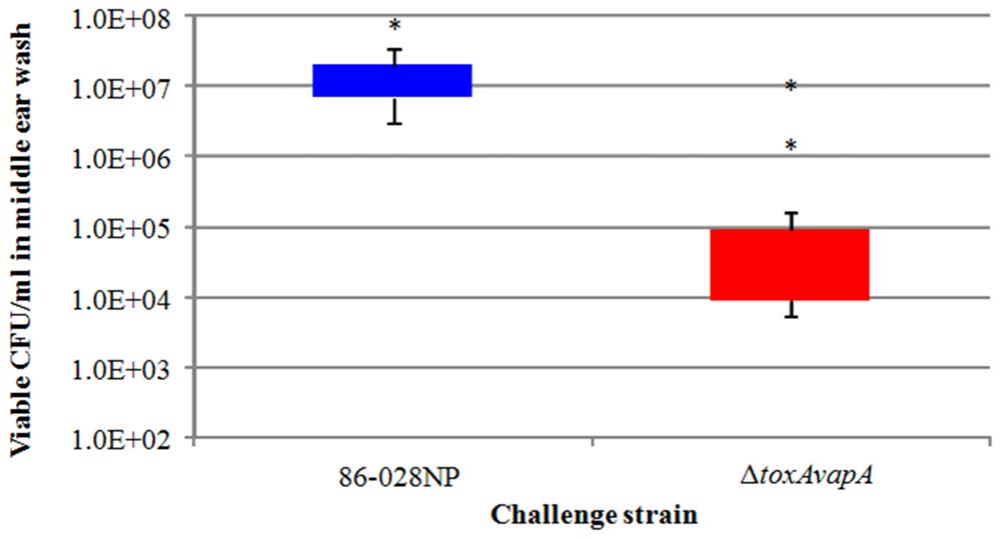
Viable wild-type and Δ*toxAvapA* mutant CFU/ml recovered from chinchilla middle ears after 4 days of infection. Asterisks denote outliers; *p* = 0.01; n = 8 ears.

Because we noted a significant difference in the ability of the ΔtoxAvapA mutant to survive during long-term co-culture in human tissues, we performed challenge studies using the chinchilla model of otitis media. For these assays, four animals (8 ears each) were inoculated through the superior bullae with either the wild-type strain or the ΔtoxAvapA mutant, with the same strain inoculated into both ears. Four days after infection, the bullae were opened and lavaged, and dilutions of the lavage fluid were drop-plated onto chocolate agar plates and incubated for 24 hours at 35°C in 5% CO_2_. Figure 2 shows a boxplot of the difference between the recovery of the mutant versus the wild-type strain during in vivo infection. Asterisks denote outliers, defined as ± 1.5× the interquartile range [19]. Similar to the in vitro results, the ΔtoxAvapA mutant displayed significantly diminished recovery from the chinchilla middle ear, indicating that this TA locus contributes to NTHi survival during infection.

**Figure 3:**
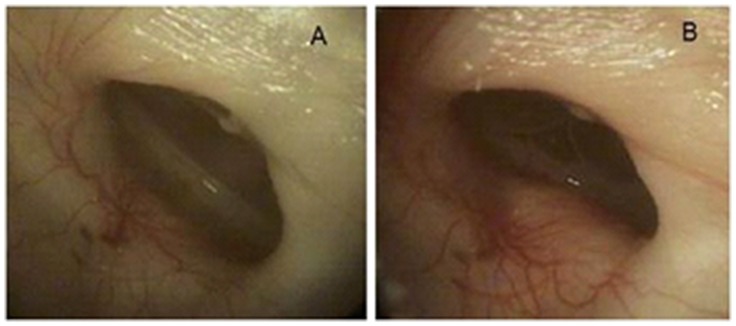
Gross otoscopic view of the tympanic membrane of a chinchilla. The same animal imaged (A) before infection; and (B) 4 days after inoculation with the Δ*toxAvapA* mutant. Edema and redness indicate a productive infection.

**Figure 4:**
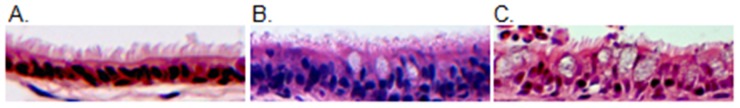
H&E-stained sections of chinchilla middle ears (400× magnification). A. Middle ear mucosa of an uninfected control animal. B. The middle ear of an animal infected with the Δ*toxAvapA* mutant for 4 days. Note the characteristic goblet cell hyperplasia associated with a productive NTHi infection. C. The middle ear of an animal infected with the wild type parent strain 86-028NP. The middle ear lumen is at the top of each micrograph.

Otoscopic images of the tympanic membrane of an animal before (A) and 4 days after inoculation with the ΔtoxAvapA mutant (B) are shown in Figure 3. Swelling and redness can be observed around the membrane, suggesting inflammation. An average of 1.5×10^5^ viable CFU/ml was obtained from a lavage of this ear. To confirm a productive infection, the bulla of this animal was step-sectioned and mounted onto slides. Images of hematoxylin-eosin (H&E)-stained sections of the middle ears of three animals are shown in Figure 4. Note the characteristic goblet cell hyperplasia and edema observed during NTHi infection (ΔtoxAvapA, 4B and wild type, 4C) compared to the uninfected control animal (4A). This indicates that the ΔtoxAvapA mutant strain was capable of producing an infection, even though it was less able to survive in vivo than the wild-type parent.

### Microarray Analysis

In order to identify any effects on the transcriptome of deleting the toxAvapA TA locus in NTHi, we performed microarray analysis on total RNA from both the wild-type and the mutant strain grown in defined media to an OD_600_ of ∼0.4. Interestingly, we found a significant (defined as greater than 2-fold) reduction in the transcription of the tryptophan biosynthesis regulon (Table 3). We confirmed this microarray data by qPCR of NTHI1763 (trpCF), a key gene in the tryptophan biosynthesis pathway. However, we did not observe significant differences between the wild-type and mutant strains in the transcription of the tryptophanase and tryptophan permease genes tnaA and tnaB (NTHI0831-0832), the tryptophanyl-tRNA synthetase trpS (NTHI0755), the Trp operon repressor trpR (NTHI0996) or the high-affinity tryptophan-specific transport protein gene mtr (NTHI0396). This suggested that transcription of the biosynthetic regulon was specifically affected. To further investigate these results, we performed HPLC studies on conditioned medium of both the wild-type and mutant strains to determine the levels of tryptophan remaining in the media. All ≥2-fold differentially-regulated genes identified in the ΔtoxAvapA mutant and their fold changes are listed in Supplemental Table 1.

**Table 3:** Fold decrease in transcription of the tryptophan biosynthesis regulon in the NTHi Δ*toxAvapA* mutant compared to the wild-type strain.

Gene	Fold Decrease	Description
NTHI1701	2.591	Tryptophan synthase alpha chain; TrpA
NTHI1702	2.150	Tryptophan synthase beta chain; TrpB
NTHI1763	6.183	Tryptophan biosynthesis protein; TrpCF
NTHI1764	3.442	Anthranilate phosphoribosyltransferase; TrpD
NTHI1767	3.574	Glutamine amidotransferase; TrpG
NTHI1768	3.068	Anthranilate synthase component I; TrpE

### HPLC of Conditioned Medium

In Escherichia coli and in many other bacteria, the regulation of the tryptophan biosynthetic operon is controlled in part by the levels of available tryptophan in the organism [20]. Therefore, we were interested in determining whether there were any differences in the relative concentrations of tryptophan remaining in conditioned medium between the wild-type and mutant strains. If there were, this would suggest that the tryptophan regulon was depressed due to an increased transport of tryptophan from the medium into the mutant strain. To ascertain this, we performed high performance liquid chromatography (HPLC) on samples of conditioned defined medium from the wild-type and mutant strains to compare the levels of tryptophan remaining in the media. We grew both organisms in defined media to an OD_600_ of ∼0.4, harvested the supernatant, pooled 3 biological replicates for each strain and passed the media through a 3 kDa filter to remove most of the larger protein and membrane components. The filtrate was then derivatized with o-phthaldialdehyde/mercapto-ethanol and run against an authentic tryptophan standard. Table 4 shows that there was no significant difference between the levels of tryptophan remaining in the supernatant of conditioned medium from the wild-type or mutant strains.

**Table 4:** HPLC results of the relative amounts of tryptophan in derivatized samples of conditioned medium from the wild-type or mutant NTHi strains.

Strain	Micrograms of tryptophan in sample (±SD)	p value
86-028NP	0.315 (0.003)	>0.05
ΔtoxAvapA	0.365 (0.049)	(not significant)

These results indicate that the observed decrease in the transcription of the tryptophan regulon in the ΔtoxAvapA mutant was not due to increased transport of this amino acid into the mutant strain. This is further supported by our findings that there were no significant differences in the transcription of genes active in the degradation or transport of tryptophan in the mutant as compared to the wild-type strain. Since HigA antitoxin homologues in other organisms have not been found to be involved in the regulation of genes other than their own operons [21], it is not clear why the NTHi ΔtoxAvapA mutation results in decreased tryptophan regulon transcription.

### RNase Activity Assays

**Figure 5:**
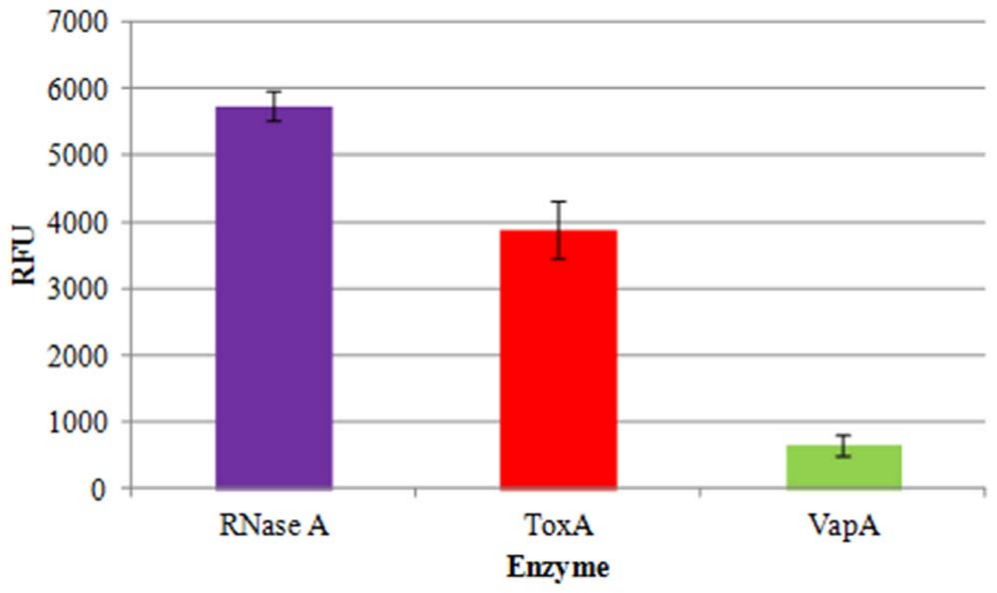
Ribonuclease activity assays using purified ToxA and VapA proteins. Eighteen pmol of ToxA, VapA and the positive control RNase A were incubated at 37°C for one hour with 5 pmol of RNaseAlert substrate. The fluorescence of the negative control (buffer alone) was subtracted from each value. (n = 3, RFU  =  relative fluorescence units).

**Figure 6:**
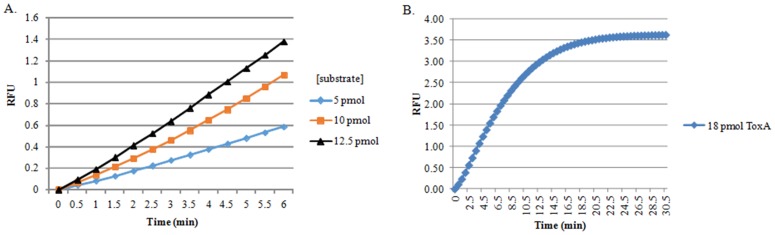
Reaction progress curves of ToxA activity. A. Initial reaction progress for the first 6 minutes measured in a MiniOpticon at 37°C with increasing concentrations of RNaseAlert substrate and 18 pmol ToxA. B. Reaction approaching completion at 30.5 minutes (5 pmol RNaseAlert substrate; 18 pmol ToxA).

To determine if the HigB homologue in NTHi could cleave free RNA, we purified both the toxin (ToxA) and antitoxin (VapA) proteins and subjected each to ribonuclease activity assays. This assay consists of a commercially-available RNA substrate that has a quencher on one end and a fluorophore (FAM) on the other (RNaseAlert®, Integrated DNA Technologies, Coralville, IA USA). The intact substrate is not fluorescent, but when cleaved it emits a bright green fluorescence. The probe is a single RNA moiety of a proprietary length and sequence. RNA cleavage is monitored by fluorescence intensity measured on the fluorescein channel (485 nm excitation, 520 nm emission) that increases over time. Figure 5 shows the results of 5 pmol of substrate incubated at 37°C for one hour with 18 pmol of purified ToxA and VapA. The negative control (protein renaturation buffer) was subtracted from each value, and the positive control was 18 pmol of commercially-available RNase A. To further characterize the ribonuclease activity of ToxA, kinetic studies were performed in which the concentration of the RNaseAlert substrate was increased while keeping the concentration of ToxA in each reaction at 18 pmol. Figure 6A illustrates the initial reaction progress in the first 6 minutes with increasing concentrations of substrate, while Figure 6B shows the reaction approaching completion at 30.5 minutes (5 pmol RNaseAlert; 18 pmol ToxA).

The HigB toxin from *P. vulgaris* has been identified as having ribonuclease activity on mRNA in the context of a ribosome at A-rich sites in *E. coli* [22], and here we show that ToxA from NTHi displays activity on RNA that is unbound in solution. This is consistent with our previous results, in which we have shown that two other TA loci toxins in NTHi, VapC-1 and VapD, are active on free RNA *in vitro* [13], [23]. Other studies have found that induced ectopic expression of the HigB toxin in *M. tuberculosis* lead to growth arrest and cell death as well as cleavage of tmRNA and mRNA predominantly from genes regulated by the IdeR iron-dependent repressor and the zinc uptake repressor Zur [24]. However, the HigB cleavage sites identified in the *M. tuberculosis* tmRNA *ssrA* in that investigation were not particularly A-rich. This suggests that there may be a broader range of RNA targets of the HigB toxin homologues in different organisms than originally thought. If this is true, the observed repression of the tryptophan biosynthetic regulon in the Δ*toxAvapA* strain might be due to the loss of the native function of ToxA on other targets, rather than directly on the regulon itself. Our data indicate that this loss does not influence growth in defined media, but significantly impacts NTHi survival during infection both *in vitro* and *in vivo*.

## Conclusions

We show that deleting the *toxAvapA* locus results in attenuation of NTHi survival during infection, both *in vitro* and *in vivo*. We purified ToxA and identified it as having ribonuclease activity, suggesting that the mechanism by which the *toxAvapA* TA locus affects persistence is to facilitate a state of dormancy via mRNA degradation. Unexpectedly, we found by microarray analysis that deleting this locus also resulted in the repression of the tryptophan biosynthetic regulon in the mutant strain. No significant difference between the ability of the wild-type and mutant strains to take up this amino acid from the media was identified. Further study is warranted to determine the exact mechanism by which the deletion of *toxAvapA* affects the regulation of this essential metabolic pathway.

## Acknowledgments

We thank Shirley A. Powell, HT (ASCP) for excellent histological assistance.

## Supporting Information

Table S1 All genes that displayed ≥2-fold change in transcription in the Δ*toxAvapA* mutant.
(DOC)
