# Supplementary material for: The ToxAvapA Toxin-Antitoxin Locus Contributes to the Survival of Nontypeable Haemophilus influenzae during Infection
Source: PLoS One. 2014 Mar 12;9(3):e91523. doi: 10.1371/journal.pone.0091523 (PMC3951411; doi:10.1371/journal.pone.0091523)
Supplement: Table S1 — All genes that displayed ≥2-fold change in transcription in the ΔtoxAvapA mutant. (DOC) [file pone.0091523.s001.doc]

**Table S1.** All genes that displayed ≥2-fold change in transcription in the Δ*toxAvapA* mutant.

| Gene | Fold Change | Description |
| --- | --- | --- |
| NTHI1025 | 2.308 up | Similar to: HI0857, YGFE_HAEIN; hypothetical protein |
| NTHI1336 | 2.212 up | Similar to: HI1168, YB68_HAEIN; hypothetical protein |
| NTHI1450 | 2.188 down | HMW2A |
| NTHI1565 | 2.587 down | Hypothetical protein |
| NTHI1566 | 3.517 down | Hypothetical protein |
| NTHI1567 | 3.223 down | Similar to: YBEQ_ECOLI; hypothetical protein |
| NTHI1569 | 2.957 down | Hypothetical protein |
| NTHI1571 | 2.427 down | Hypothetical protein |
| NTHI1701 | 2.591 down | Tryptophan synthase alpha chain; TrpA |
| NTHI1702 | 2.150 down | Tryptophan synthase beta chain; TrpB |
| NTHI1723 | 2.011 down | Hypothetical protein |
| NTHI1762 | 3.765 down | Hypothetical protein |
| NTHI1763 | 6.183 down | Tryptophan biosynthesis protein; TrpCF |
| NTHI1764 | 3.442 down | Anthranilate phosphoribosyltransferase; TrpD |
| NTHI1765 | 4.290 down | Similar to: HI1388.1, YD8A_HAEIN; hypothetical protein |
| NTHI1767 | 3.574 down | Glutamine amidotransferase; TrpG |
| NTHI1768 | 3.068 down | Anthranilate synthase component I; TrpE |
| NTHI1852 | 2.063 down | Hypothetical protein |
| NTHI1912 | 9.569 down | Similar to: HI1251, YC51_HAEIN; VapA |
